# Alterations in the intestinal microbiome and mental health status of workers in an underground tunnel environment

**DOI:** 10.1186/s12866-020-02056-3

**Published:** 2021-01-06

**Authors:** Zhen-Hua Lu, Yi-Wen Liu, Zhao-Hua Ji, Ting Fu, Min Yan, Zhong-jun Shao, Yong Long

**Affiliations:** 1 Department of Epidemiology, Ministry of Education Key Lab of Hazard Assessment and Control in Special Operational Environment, School of Public Health, Air Force Medical University, Xi’an, 710032 People’s Republic of China; 2 Wuwei Municipal Center for Disease Control and Prevention, Wuwei City, Gansu Province People’s Republic of China

**Keywords:** Underground tunnel environment, 16S rRNA, Gut microbiome, Mental distress, Brain-gut-microbiota axis

## Abstract

**Background:**

Working in an underground tunnel environment is unavoidable in professions such as miners and tunnel workers, and there is a concern about the health of these workers. Few studies have addressed alterations in the intestinal microbiome of workers within that environment.

**Results:**

Fecal samples were collected from the workers before they entered the tunnel (baseline status, BS) and after they left the tunnel (exposed status, ES), respectively (a time period of 3 weeks between them). We analyzed 16S rRNA sequencing to show the changes in microbial composition and self-evaluation of mental health questionnaire was also performed. The results showed that Shannon and Simpson indices decreased significantly from BS to ES. A higher abundance was found in the phylum *Actinobacteria*, classes *Actinobacteria* and *Deltaproteobacteria*, orders *Bifidobacteriales*, *Coriobacteriales*, and *Desulfovibrionales*, families *Bifidobacteriaceae*, *Peptostreptococcaceae*, *Coriobacteriaceae*, *Clostridiaceae_1*, *Desulfovibrionaceae*, Pseudomonadaceae, and Microbacteriaceae, and genera *Bifidobacterium*, *Romboutsia*, *Clostridium* sensu stricto, and *Leucobacter* in ES, while BS showed greater levels of genera *Faecalibacterium* and *Roseburia*. The self-evaluation showed that at least one-half of the tunnel workers experienced one or more symptoms of mental distress (inattention, sleeplessness, loss of appetite, headache or dizziness, irritability) after working in the underground tunnel environment.

**Conclusions:**

Collectively, the underground tunnel environment led to alterations in the intestinal microbiome, which might be relevant to symptoms of mental distress in underground-tunnel workers.

## Background

In the underground working environment, several agents, such as noise, vibration, temperature, humidity, dust, chemical fumes, and radon could influence the health and safety of workers [1, 2]. In addition, the highly intense working pressure over a long period of time has a tendency to adversely affect the health of these workers [3]. Surveys have shown that miner mortality from lung cancer is significantly higher than that for the general population [4]. That mortality also differed greatly between underground miners and surface miners [4]. Furthermore, it is known that underground workers suffer a series of mental health problems. Joaquim [5] has investigated that depression, light or moderate anxiety, and a decrease in the quality of sleep interrupts the health of underground coal miners in southern Brazil. One research in China has studied anxiety and depression in coal miners and their related influencing factors [6]. Another study among 2500 Chinese underground coal miners revealed that the prevalence of symptoms of depression was 62.8% and was significantly associated with an imbalance between high effort and reward, the perceived physical environment, work–family conflict, and overcommitment [7].

Mounting evidence in recent years has suggested a close relationship between the intestinal microbiome in humans and their health. There are approximately 100 trillion microbes in the human gut, which is 10-fold more than the number of total cells in the human host. The stable and healthy microenvironment in humans is dynamically maintained by the microbes’ millions of nonredundant genes. Accordingly, dysbiosis in intestinal flora has been suggested to play a vital role in the pathogenesis of adverse systemic conditions, such as metabolic [8], inflammatory [9], immune [10], and neuropsychiatric disorders [11].

To our knowledge, the literature is limited on the alteration of the intestinal microbiome in underground tunnel workers. The aim of the current study was to provide new insights into the health of these workers by probing into the changes in their intestinal microbiomes. We also investigated the changes in mental health of these workers after working for 3 weeks in the tunnel environment to help shed new light on a potential strategy by which to assess their health and prevent and control the various diseases associate with this type of work.

## Results

### Demographics and subject characteristics

Demographic characteristics of the study subjects are presented in Table 1. According to the results of the self-evaluation using the questionnaire (Table 2), all 48 workers experienced frequent or occasional inattention, and most experienced periods of sleeplessness. Appetite loss occurred in 61.3% of the workers, headache or dizziness in 54%, and irritability in nearly 50%.

**Table 1:** Demographic characteristics of study subjects

Variable	Case	Percentage
Gender		
male	48	100%
female	0	0%
Age (years)		
≤ 20	7	14.6%
21–30	31	64.6%
31–40	9	18.8%
> 40	1	2.1%
Education		
Junior high school or under	5	10.4%
Senior high school/technical secondary school	20	41.7%
Junior college or above	23	47.9%
Marital status		
Single/divorced/widowed/separated	36	75.0%
Married	12	25.0%
Reproductive history		
parous	10	20.8%
nonparous	38	79.2%
Smoke		
yes	25	52.1%
no	18	37.5%
quit smoking(> 1 year)	5	10.4%

**Table 2:** Self-evaluation using the overall health questionnaire

Symptom	Case	Percentage
Dizziness/Headache		
frequently	1	2.08%
occasionally	26	54.17%
never	21	43.75%
Inattention		
frequently	21	27.08%
occasionally	54	72.92%
never	0	0.00%
Sleeplessness		
frequently	6	12.50%
occasionally	28	58.33%
never	14	29.17%
Inappetence		
frequently	4	8.33%
occasionally	26	54.17%
never	18	37.50%
Irritability		
frequently	3	6.25%
occasionally	22	45.83%
never	23	47.92%

### Microbial diversity analysis

**Fig. 1:**
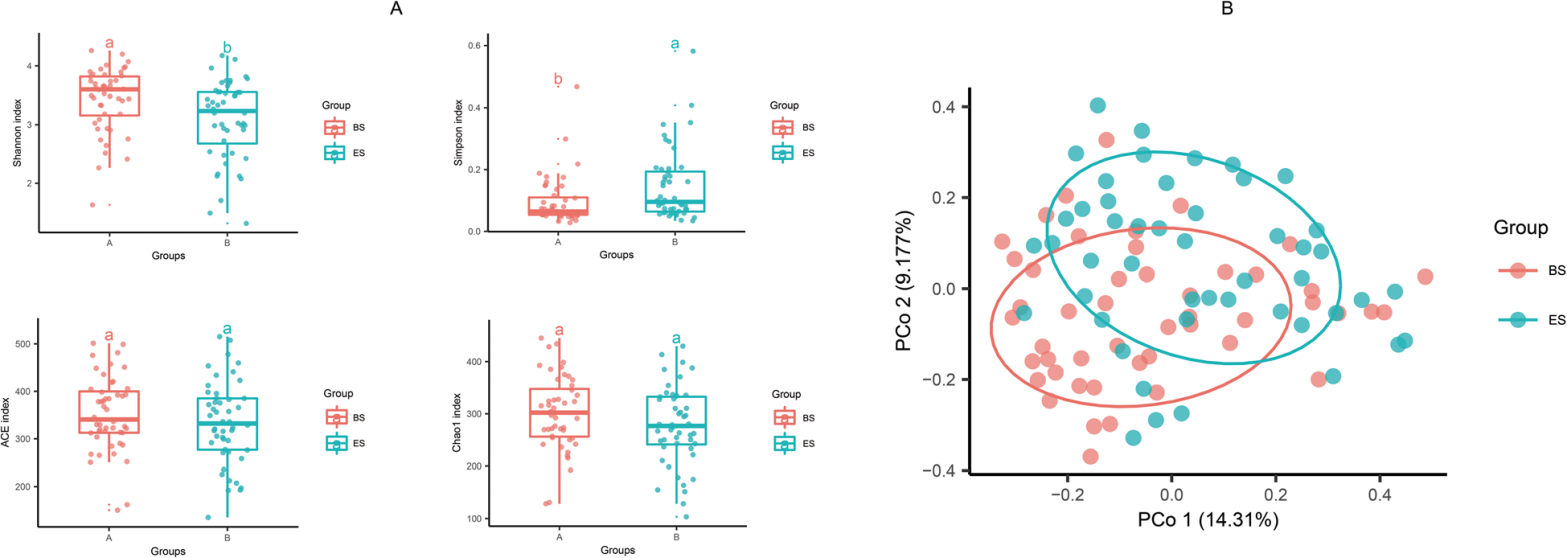
**a** Microbial alpha diversity in ES and BS. Boxplot showed greater gut microbial diversity in BS than ES according to Shannon (t = 3.375, *P* = 0.001) and Simpson (t = 2.757, *P* = 0.008) indices with paired samples t-test. **b** Microbial beta diversity. Principal coordinate analysis (PCoA) of ES compared to BS based on Bray-Curtis dissimilarities in the relative abundance of ASVs (PERMANOVA, *R*^2^ = 0.028, *P* = 0.002)

Statistical analyses of the 16S rRNA sequences showed 1272 ASVs. The results of the alpha diversity analyses were quantified using the Shannon, Simpson, Ace, and Chao1 indices (Fig. 1a). Gut microbial diversity, which was estimated using the Shannon (t = 3.375, *P* = 0.001) and Simpson (t = 2.757, *P* = 0.008) indices, was significantly lower at ES compared to that at BS; however, Chao1 (t = 1.946, *P* > 0.05) and Ace (t = 1.898, *P* > 0.05) indices used to estimate richness showed a trend similar to that of the Shannon and Simpson indices but without statistical significance.

Beta diversity was evaluated at the ASV level. To compare the composition of the identified bacterial community members within the workers and identify the main factors driving that community composition, a Bray–Curtis dissimilarity matrix was calculated using the data on normalized and square-root transformed read abundance. Overall similarities in the structure of the bacterial community among the samples were displayed using PCoAs. PCoA analyses revealed strong clustering of bacterial communities which is statistically significant with PERMANOVA test (*R*^2^ = 0.028, *P* = 0.002) according to the two groups at the ASV level, in which 14.31% of the total principal component 1 and 9.177% comprised principal component 2 (Fig. 1b).

### Structural analysis of the bacterial community

**Fig. 2:**
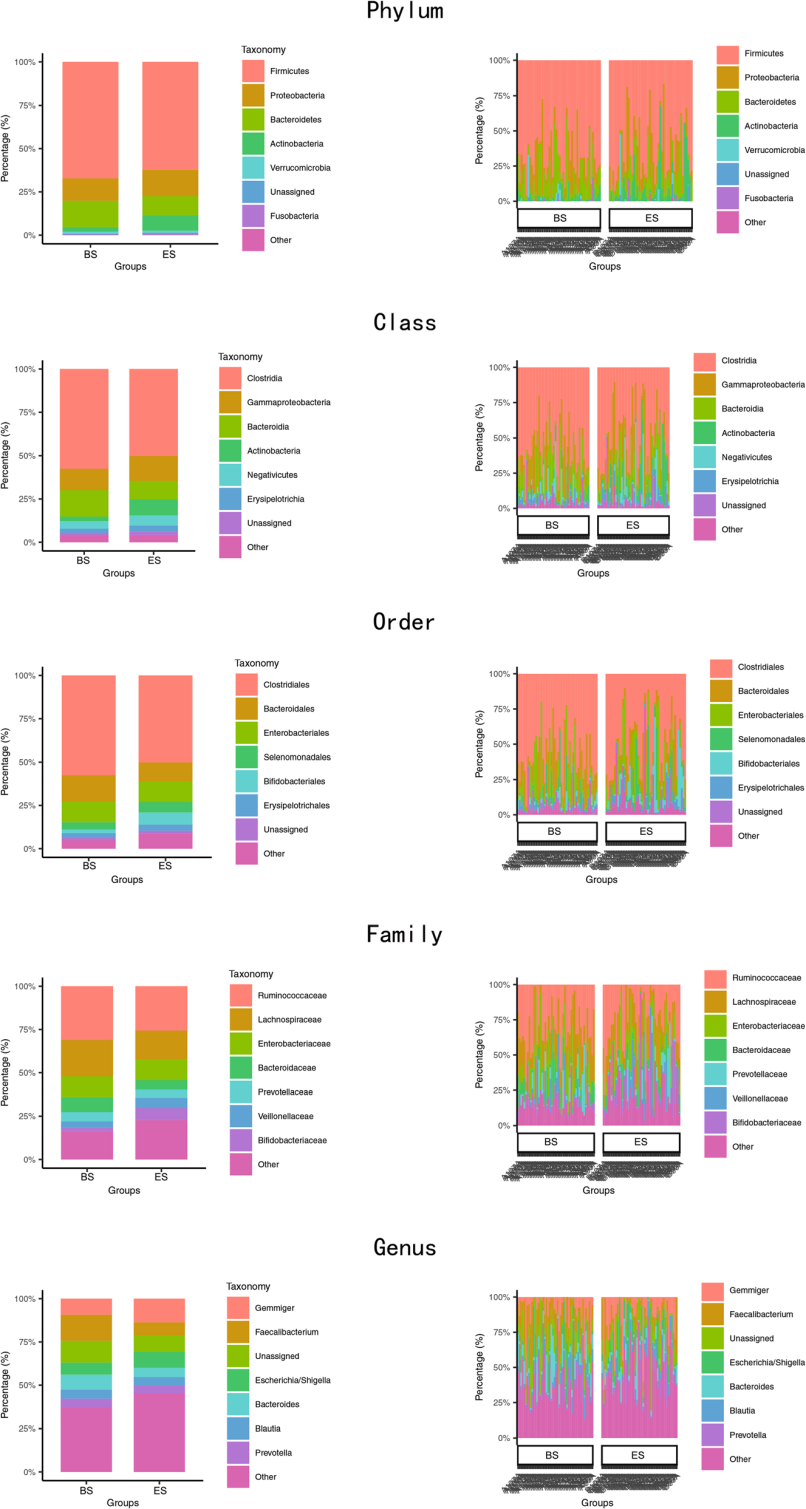
Bacterial community structural composition and distribution. Dominant bacterial composition detected in each sample and two status were showed with overlapping histogram at five taxonomical levels (phylum, class, order, family, genus)

At the phylum level, the dominant flora at BS belonged mainly to *Firmicutes* (67.18%), *Bacteroidetes* (15.30%), and *Proteobacteria* (12.88%) and accounted for 95.36% of the total bacteria; 4.64% comprised other bacteria (Fig. 2). Compared to that at BS, the predominant flora at ES showed little change. The total bacteria comprised *Firmicutes* (62.23%), *Proteobacteria* (15.23%), and *Bacteroidetes* (10.90%), or 88.36% (Fig. 2). Importantly, the *Actinobacteria* increased from 2.67% at BS to 9.07% at ES. The other four levels were also displayed in Fig. 2.

**Fig. 3:**
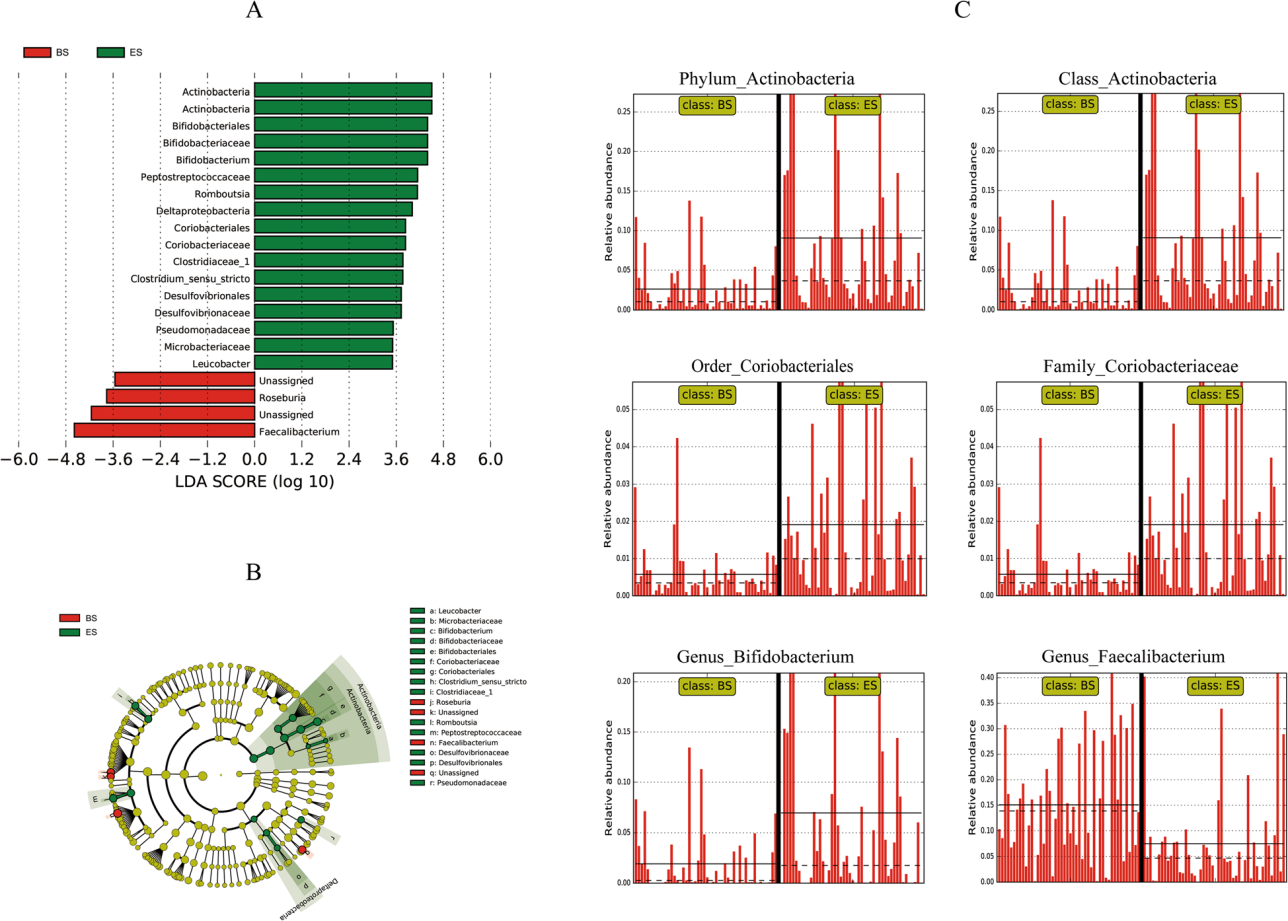
LEfSe and LDA analysis revealed changes in the taxonomic composition of the gut microbiota in ES compared to BS tunnel workers. **a** LDA scores showing the biomarker taxa (LDA score of > 3.5 and a significance of *P* < 0.05 determined by the Wilcoxon signed-rank test) for ES (red) and BS (green). **b** Cladogram showing the relationship between taxon (the levels represent, from the inner to outer rings, phylum, class, order, family, and genus) in ES (red) and BS (green). **c** Difference features histogram in a panel showed relative abundance of mental disorders-related biomarker taxa. The straight and dotted lines plot means and medians of the relative abundance, respectively, in each subgroup

LEfSe was used to identify differential microbial abundances between BS and ES (Fig. 3). This analysis revealed significant differences (LDA ≥ 3.5, *P* < 0.05 determined by the Wilcoxon signed-rank test) in bacterial clades from phylum to genus levels between BS and ES. Increased *Actinobacteria* (LDA = 4.50, *P* = 0.001) was observed at ES at the phylum level. Analysis at the class level also showed elevated levels of *Actinobacteria* (LDA = 4.50, *P* = 0.001) at ES. At the order level, *Bifidobacteriales* (LDA = 4.39, *P* = 0.041), *Coriobacteriales* (LDA = 3.83, *P* = 0.009), and *Desulfovibrionales* (LDA = 3.73, *P* = 0.001) increased at ES. At the family level, *Bifidobacteriaceae* (LDA = 4.39, *P* = 0.041), *Peptostreptococcaceae* (LDA = 4.14, *P* = 0.001), *Coriobacteriaceae* (LDA = 3.83, *P* = 0.009), *Clostridiaceae_1* (LDA = 3.77, *P* = 0.009), *Desulfovibrionaceae* (LDA = 3.73, *P* = 0.001), *Pseudomonadaceae* (LDA = 3.52, *P* = 0.012), and *Microbacteriaceae* (LDA = 3.51, *P* = 0.042) increased at ES. At the genus level, *Bifidobacterium* (LDA = 4.39, *P* = 0.047), *Romboutsia* (LDA = 4.14, *P* = 0.001), *Clostridium* sensu stricto (LDA = 3.77, *P* = 0.010), and *Leucobacter* (LDA = 3.50, *P* = 0.042) significantly increased at ES, and *Faecalibacterium* (LDA = 4.59, *P* = 0.001), and *Roseburia* (LDA = 3.76, *P* = 0.043) levels were higher at BS than at ES.

## Discussion

**Fig. 4:**
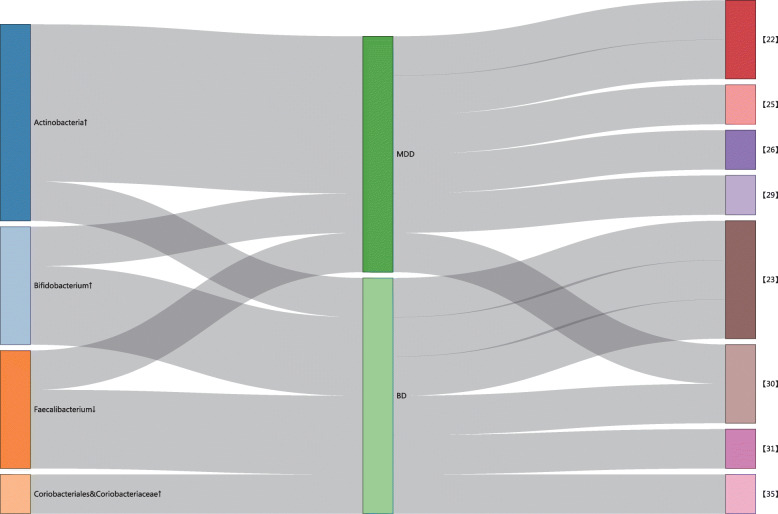
Sankey diagram of the consistency in current study with published literatures. Left side mainly showed the changes of four critical intestinal microbiome in both current study and published literatures. Middle and Right side represented the result of mood disorder in specific literature

In our current research, alterations in the intestinal microbiome of workers was determined before and after 3 weeks of continuously working in underground tunnels. Moreover, because studies have demonstrated that underground-tunnel workers suffer symptoms of a series of mental health disorders, this relationship was also investigated. The results showed that microbiome diversity at ES was significantly different from that at BS. Surprisingly, we found that alterations in the genera of the intestinal microbiome in these tunnel workers were similar to those in patients with mental disorders (Fig. 4), especially mood disorders [12]. Study has shown that brain activity can be affected by the intestinal microbiome through the brain–gut–microbiota axis [13]. In addition, Kelly [14] has demonstrated that alteration of the composition of the intestinal microbiome in depression patients is associated with dysbiosis in the function of the hypothalamic–pituitary–adrenal (HPA) axis, intestinal low-grade inflammation, and an imbalanced neurotransmitter metabolism through the brain–gut–microbiota axis. This information may provide a potential for treatment and prevention of mental distress experienced by underground workers.

Our study found that the diversity of the intestinal microbiome at BS was greater than that at ES. Gut microbiota diversity could be affected by various factors, such as different dietary patterns, race, age, and environment [15, 16]. Researchers have proved that alpha diversity is potentially correlated with the pathogenesis of several mental diseases. For example, a lower bacterial diversity is found to be related to anorexia nervosa [17]. Another study found a greater diversity of intestinal microbiome in healthy people compared with that in patients with bipolar depression [18], which is consistent with the results of our current study. Conversely, Jiang et al. [19] have found increased alpha diversity in patients with acute major depression compared with that in healthy individuals. Painold [20] has shown that alpha diversity is negatively correlated with illness duration in individuals with bipolar depression; however, some studies have reported no significant difference in gut microbiota diversity between patients with major depressive disorder (MDD) and healthy individuals [21–23].

Our study showed significant differences in the amount of Actinobacteria, order Coriobacteriales, and family Coriobacteriaceae between the two groups, which were consistent with Painold [20] in the study of patients with bipolar disorder (BD). Actinobacteria, Coriobacteriales, and Coriobacteriaceae are commonly known to be associated with lipid metabolism [24] and related to cholesterol levels [25], which may suggest that elevated abundance of Actinobacteria and Coriobacteriaceae in patients with mental disorders is possibly correlated to dyslipidemia. The result of elevated Actinobacteria at ES was also in line with Jiang et al. [19], Zheng et al. [22], Chen et al. [23], and Chung et al. [26] in patients with MDD.

*Bifidobacterium* was significantly higher in workers after working in the tunnel for 3 weeks. This finding is consistent with that of Rong et al. [27] and Guo et al. [28]. Rong et al. found that the relative abundance of *Bifidobacterium* is significantly higher in patients with MDD and BD with current major depressive episodes. Similarly, Guo et al. found that the relative abundances of *Bifidobacterium* increases in manic individuals with BP; however, a Japanese study has found lower *Bifidobacterium* counts in patients with MDD [29]. This may be because of the differences in the races studied. As a probiotic, *Bifidobacterium* was considered beneficial for the human body because it also synthesizes gamma aminobutyric acid (GABA), a known predominant inhibitory neurotransmitter in the nervous system, which could regulate the immunological [30] and neurophysiological [31] processes using a receptor-mediated pattern. The increased GABA-synthesizing bacteria in gut of workers may verify the presence of mental distress symptoms.

Another important finding from the current study was the group difference in the amount of *Faecalibacterium*. Several studies have demonstrated lower *Faecalibacterium* levels in patients with mental disorders, including BP [20, 32] and MDD [19] than in healthy individuals. These results have identified a decrease in *Faecalibacterium* as a discriminating feature in mood disorders and added strength to our current results. According to Evans [32], the lower genera levels may be native to the illness and relevant to a depressed state. Noteworthy, *Faecalibacterium* has been shown to be associated with anti-inflammatory activity both in vitro and in vivo [33, 34]. As a Gram-positive butyrate-producing gut bacterium, *Faecalibacterium* is considered to be a vital genus associated with the short chain fatty acid (SCFA)–producing pathway [35]. The decrease in SCFA-related bacteria in our study may indicate dysbiosis on the anti-inflammatory activities in the workers. Furthermore, SCFAs could directly or indirectly mediate microbiota–gut–brain interactions through signaling pathways, including the immune, endocrine, neural, and humoral routes [36], which strengthens our hypothesis.

There were some limitations of our current study. The sample size was insufficient and additional research will be needed using a larger sample size. In addition, all the workers in this study were male; therefore, the influence of sex on the results could not be estimated. Moreover, the effects of type of work, working hours, eating habits, alcohol consumption, smoking, and lifestyles on the intestinal flora were not properly considered.

## Conclusions

Working in an underground environment greatly influences the mental health and intestinal microbiome of workers. Moreover, changes of their intestinal microbiome are similar to those seen in patients with mental disorders, which may throw light that the intestinal microbiome is relevant to mental health of tunnel workers. This study is the first to investigate changes in the intestinal microbiomes of workers in an underground tunnel setting, and its findings may be useful for prevention and treatment of mental health disorders among tunnel workers.

## Methods

### Study participants

Our study comprised 48 healthy subjects who were recruited as tunnel workers from northwest China and who participated in an integrated washout period for 2 weeks before entering the underground environment. All subjects were males, 19–43 years old (mean = 26.02 years, SD = 5.65 years). Ninety-six fecal samples were collected from the 48 tunnel workers before and after 3 weeks of working in the tunnel (8 h per day for work) to determine a baseline status (BS) and exposed status (ES), respectively. A standardized diet (the yogurt with probiotics, high-sugar, high-protein and high-fat food were excluded) was provided during the washout and exposed periods. And the use of antibiotics has been strictly limited. Informed consent was obtained from all participants. The fecal specimens used for microbiota analyses were frozen immediately after sampling and stored at − 20 °C.

### Questionnaire

Before entering the tunnel, data on baseline profiles and history were collected to understand the health status of all the workers studied. A self-evaluation using a mental health questionnaire was immediately completed with help of trained professionals after 3 weeks the workers left the tunnel.

### DNA extraction

Total DNA was extracted from the fecal samples using the TIANamp Micro DNA Kit (TIANGEN, Beijing, China) according to the manufacturer’s instructions. The samples were centrifuged at 1800×g for 5 min at 4 °C and the supernatant was removed from the pellet. The eluted DNA concentration was determined at an absorbance of 260 nm (A260), and purity was estimated by determining the A260/A280 value using the Nanodrop 2000c spectrophotometer (Thermo Scientific, Wilmington, USA).

### 16S rRNA library preparation and DNA sequencing

The V4–V5 regions of the bacteria 16S ribosomal RNA gene were amplified using polymerase chain reaction (PCR) as follows: 95 °C for 2 min, followed by 25 cycles at 95 °C for 30 s, 55 °C for 30 s, 72 °C for 30 s, and a final extension at 72 °C for 5 min using primers 515F 5′-barcodeTGCCAGCMGCCGCGG-3′ and 907R 5′-CCGTCAATTCMTTTRAGTTT-3′, where barcode is an eight-base sequence unique to each sample. PCR was conducted in triplicate using a 20-μL mixture containing 4 μL 5 x FastPfu Buffer, 2 μL 2.5 mM dNTPs, 0.8 μL each primer (5 μM), 0.4 μL FastPfu Polymerase, and 10 ng template DNA. Amplicons were extracted from 2% agarose gels and purified using the AxyPrep DNA Gel Extraction Kit (Axygen Biosciences, Union City, CA, USA) according to the manufacturer’s instructions and quantified using QuantiFluor™ -ST (Promega Corporation, Madison, WI, USA). Purified PCR products were quantified using Qubit®3.0 (Life Invitrogen, USA) and every 24 amplicons having different barcodes were mixed equally. The pooled DNA product was used to construct an Illumina pair-end library following Illumina’s procedures. The amplicon library was then paired-end sequenced (2 × 250) using the Illumina Hiseq platform (CapitalBio Technology Co., Ltd., Beijing, China) according to standard protocols. Primers and barcodes were removed. Each sample had two forward and reverse sequencing read data files.

### Sequence processing and analyses

#### Quality filtering and merging

Raw fastq files were quality-filtered and pair-merged using VSEARCH ver. 2.14.1 [37]. Truncation of sequence reads not having an average quality of 20 over a 30-bp sliding window and trimmed reads with < 75% of their original length as well as its paired reads were removed. The reads contaminated by adapters (default parameter: 15 bases overlapped by reads and adapter with a maximum of a 3-base mismatch allowed), the reads with an ambiguous base (N base) and its paired reads, and the reads with low complexity (10 consecutive same bases) were removed.

#### Dereplication, denoising, and chimera detecting

After merging the paired reads, dereplication, denoising, and chimera checking were performed using VSEARCH. Specifically, the reads were dereplicated using the “derep_fulllength” function in VSEARCH with a minimum unique group size set to 10 to determine unique read sequences and abundances. The reads were denoised using the UNOISE3 arithmetic command to yield amplicon sequence variants (ASVs) and information on their abundance. VSEARCH was then used to remove chimeras both de novo and compared to the ribosomal database project (RDP Release 11) classifier training reference. Each sequence was annotated with a species classification using the RDP classifier with VSEARCH.

#### Microbial diversity analysis

Alpha and beta diversity analyses were performed using USEARCH ver. 10.0.240 [38]. Alpha diversity was analyzed based on Shannon, Simpson, Ace, and Chao1 indices, and then a paired samples *t*-test was used to detect whether the index value between the two groups was significantly different. Beta diversity was analyzed with the permutational multivariate analysis of variance (PERMANOVA) based on the Bray–Curtis dissimilarity matrices. R (https://github.com/microbiota) was used for visualizing diversity analysis. Principal coordinates analyses (PCoAs) were also conducted using R to visualize the dissimilarity matrix for all samples, such that samples that were more similar could be closer in space than those that were more divergent.

#### Bacterial community structural analysis

To compare the changes in ASVs, the data on the two groups were first analyzed using R language. To determine the causes of the differences among the bacterial communities, the community structure at different taxonomic levels (i.e., phylum, class, order, family, and genus) were summarized using USEARCH. The composition distribution of each sample and the two groups were used to create an overlapping histogram at the five taxonomic levels using R. Using linear discriminant analysis (LDA) ≥ 3.5, LEfSe analysis was conducted to detect potential bacterial biomarkers using the online galaxy server (https://huttenhower.sph.harvard.edu/galaxy/), and the LDA scores derived from LEfSe analyses were used to show the relationship among taxa using a cladogram (circular hierarchical tree) of significantly increased or decreased bacterial taxa in the microbiota between the two groups.

### Statistical analyses

The data were analyzed using SPSS ver. 26.0 (IBM Corp., Armonk, NY, USA) with the means ± standard errors of the means (SEM) and R language 3.6.1. The data on the normal distribution such as age, was measured using the mean value dependent standard deviation. A paired sample t-test was performed to evaluate the difference of alpha diversity between two groups. The permutational multivariate analysis of variance (PERMANOVA) was used with PCoA based on the Bray–Curtis dissimilarity. The Kruska–Wallis rank sum test was Size used with LEfSe analysis.

## Data Availability

The data of this study are available from the corresponding author upon request.

## Abbreviations

BS: Baseline status
ES: Exposed status
ASVs: Amplicon Sequence Variants
PCoA: Principal co-ordinates analysis
LDA: Linear discriminant analysis
HPA: Hypothalamic–pituitary–adrenal
MDD: Major depressive disorder
BD: Bipolar disorder
GABA: Gamma aminobutyric acid
SCFA: Short chain fatty acid
SD: Standard Deviation
RDP: Ribosomal database project
LEfSe: Linear discriminant analysis Effect Size
PERMANOVA: The permutational multivariate analysis of variance
